# Case Report: Discordant genetic findings in miscarriage tissue following transfer of euploid embryos

**DOI:** 10.3389/frph.2026.1758427

**Published:** 2026-01-30

**Authors:** Haiyan Bai, Chunxi Zhang, Juanzi Shi

**Affiliations:** Assisted Reproduction Center, Northwest Women’s and Children’s Hospital, Xi’an, China

**Keywords:** copy number variation, mosaicism, natural miscarriage, next-generation sequencing, Preimplantation Genetic Testing for Aneuploidy

## Abstract

**Background:**

Even after the transfer of euploid embryos selected by Preimplantation Genetic Testing for Aneuploidy (PGT-A), clinical pregnancies may still result in miscarriage. The subsequent detection of chromosomal copy number variations (CNVs) in the products of conception in some cases reflects the inherent limitations of PGT-A technology.

**Case report:**

Two couples underwent PGT-A treatments at assisted reproduction center of Northwest Women's and Children's Hospital, with the respective indications of recurrent pregnancy loss and advanced maternal age. For both, ovarian stimulation was conducted by an antagonist protocol, followed by intracytoplasmic sperm injection (ICSI). The embryos were cultured to the blastocyst stage, at which timepoint trophectoderm biopsy was performed. Following genetic analysis, available euploid blastocysts were identified for both couples. However, both pregnancies were identified as missed abortions during the first trimester after transferring one euploid blastocyst each. Subsequent CNV analysis carried out on the products of conception showed the presence of embryonic mosaicism.

**Conclusion:**

The two cases highlight the critical need for thorough patient counselling regarding the technical constraints as well as the potential risks of PGT-A. Additionally, they emphasize the indispensable value of prenatal diagnosis after transfer of euploid blastocyst identified by PGT-A.

## Introduction

Preimplantation Genetic Testing for Aneuploidy (PGT-A) is indicated for couples undergoing assisted reproductive technology (ART) who are at increased risk of embryo aneuploidy (1). This includes women of advanced maternal age, individuals with a history of unexplained recurrent pregnancy loss or repeated implantation failure, and those concurrently undergoing PGT for structural rearrangements (PGT-SR) or monogenic disorders (PGT-M). The procedure involves the genetic analysis of trophectoderm biopsies taken out of *in vitro* cultured blastocysts. The aim of PGT-A is to choose and transfer embryos identified as euploid in order to increase the chance of achieving a successful pregnancy and to reduce the possibility of miscarriage. Ultimately this enhances ART efficiency overall. Even with the transfer of an embryo screened euploid via PGT-A, approximately 10% of clinical pregnancies may still result in miscarriage, with the rate varying by patient populations (2). Due to the multifactorial etiology of miscarriage, of which PGT-A only addresses the chromosomal component, it's generally advised to carry out a thorough investigation for other potential causes after a pregnancy loss. In some instances, patients would ask for genetic testing of miscarriage tissue, which may detect abnormalities unexpectedly. The following two cases will illustrate this.

## Case description

**Figure 1:**
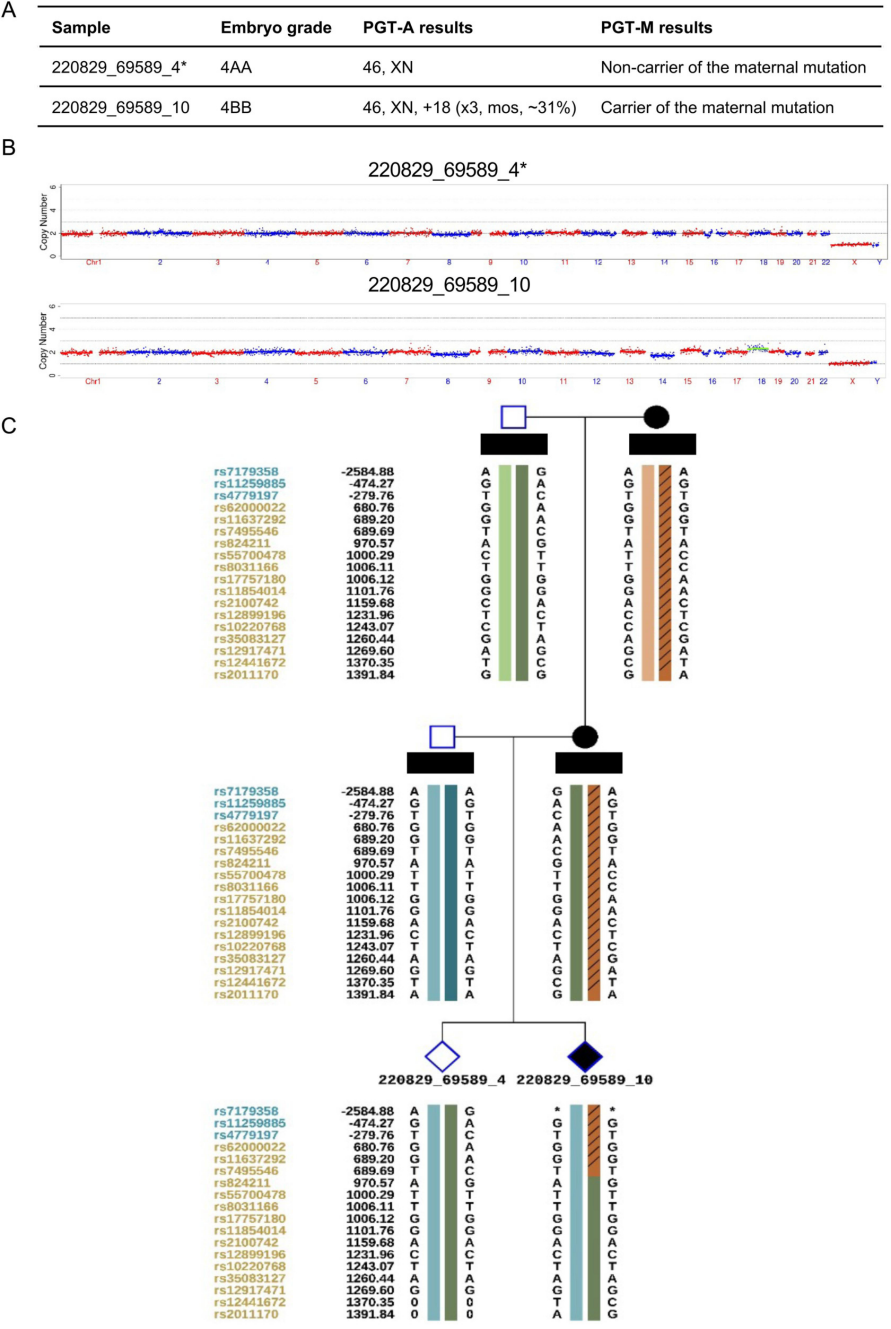
Genetic testing results of embryo and conduction of miscarriage in case 1. **(A)** PGT-M molecular genetic testing report for monogenic disorders (chromswift®). **(B)** Embryonic chromosomal copy number variations (CNVs) analysis profile. **(C)** Embryonic single nucleotide polymorphism (SNP) genotyping report. * The embryo “220829_69589_4” exhibits a low-level mosaic monosomy 8 (10%). According to the reporting criteria implemented at our reproductive center, mosaic levels below 30% are classified and reported as euploid rather than mosaic.

**Figure 2:**
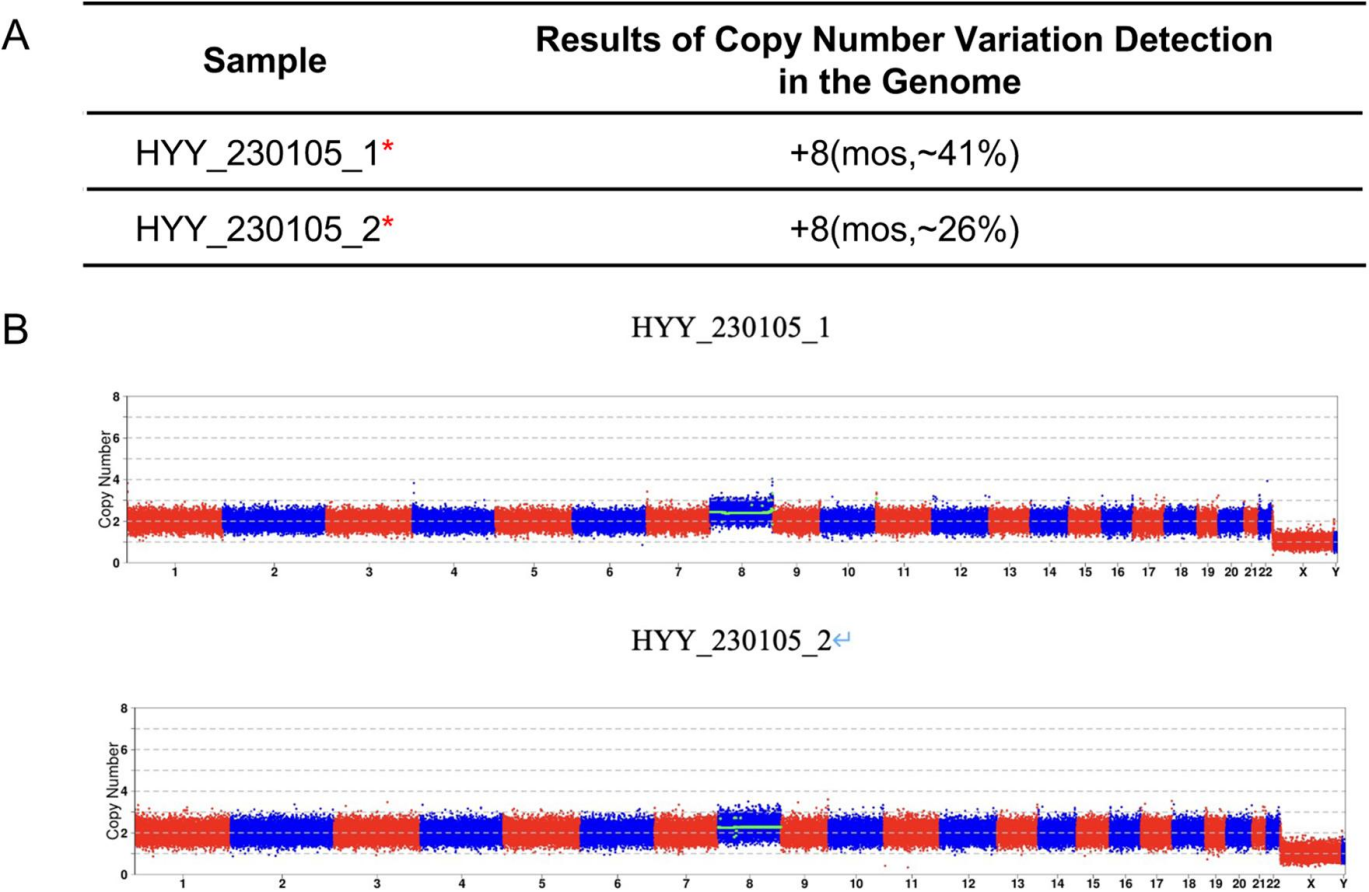
Genetic analysis of abortion tissue from case 1. **(A)** Chromosomal copy number variations (CNVs) analysis report of abortion tissue. **(B)** CNVs analysis profile of abortion tissue.

Case 1: A 31-year-old female presented with a history of three first-trimester miscarriages from 2019 to 2021. Chromosomal copy number variation (CNV) analysis was performed on the products of conception of the last miscarriage. A 300 Kb pathogenic deletion on the long arm of chromosome 15 which was maternally inherited with incomplete penetrance was detected. The tests for repeated miscarriage showed no other abnormalities. In August 2022, this patient underwent Preimplantation Genetic Testing for Monogenic Disorders and Aneuploidy (PGT-M + A) in our center for the indications of a maternal pathogenic microdeletion and recurrent pregnancy loss. using the antagonist protocol, ICSI fertilization of the 12 obtained oocytes yielded two blastocysts. The universal library prep kit (XK-013-48, XK Company) was used for DNA amplification and library preparation. Sequencing was performed with a universal gene sequencing kit (semiconductor sequencing method, XK-0159, XK Company). The results of PGT-A + M showed that there was one transferable euploid blastocyst with no deletion and one non-transferable mosaic carrier blastocyst (Figure 1). Before preparing the endometrium for FET, the female was required to undergo a serum hCG test to exclude a pre-existing pregnancy. On November 21, 2022, the euploid, non-carrier blastocyst was thawed and transferred. Serum hCG level was 456.5 mIU/mL at 12 days post-transfer. However, a transvaginal ultrasound at day 51 of gestation revealed an intrauterine gestational sac containing an embryo without any cardiac activity. The absence of fetal heartbeat was confirmed one week later. Following uterine evacuation, CNV analysis of the products of conception indicated trisomy 8 mosaicism at an approximate level of 41%. To rule out maternal cell contamination, a second sample taken from a different part of the villus tissue was reanalyzed and confirmed about 26% trisomy 8 mosaicism (Figure 2).

**Figure 3:**
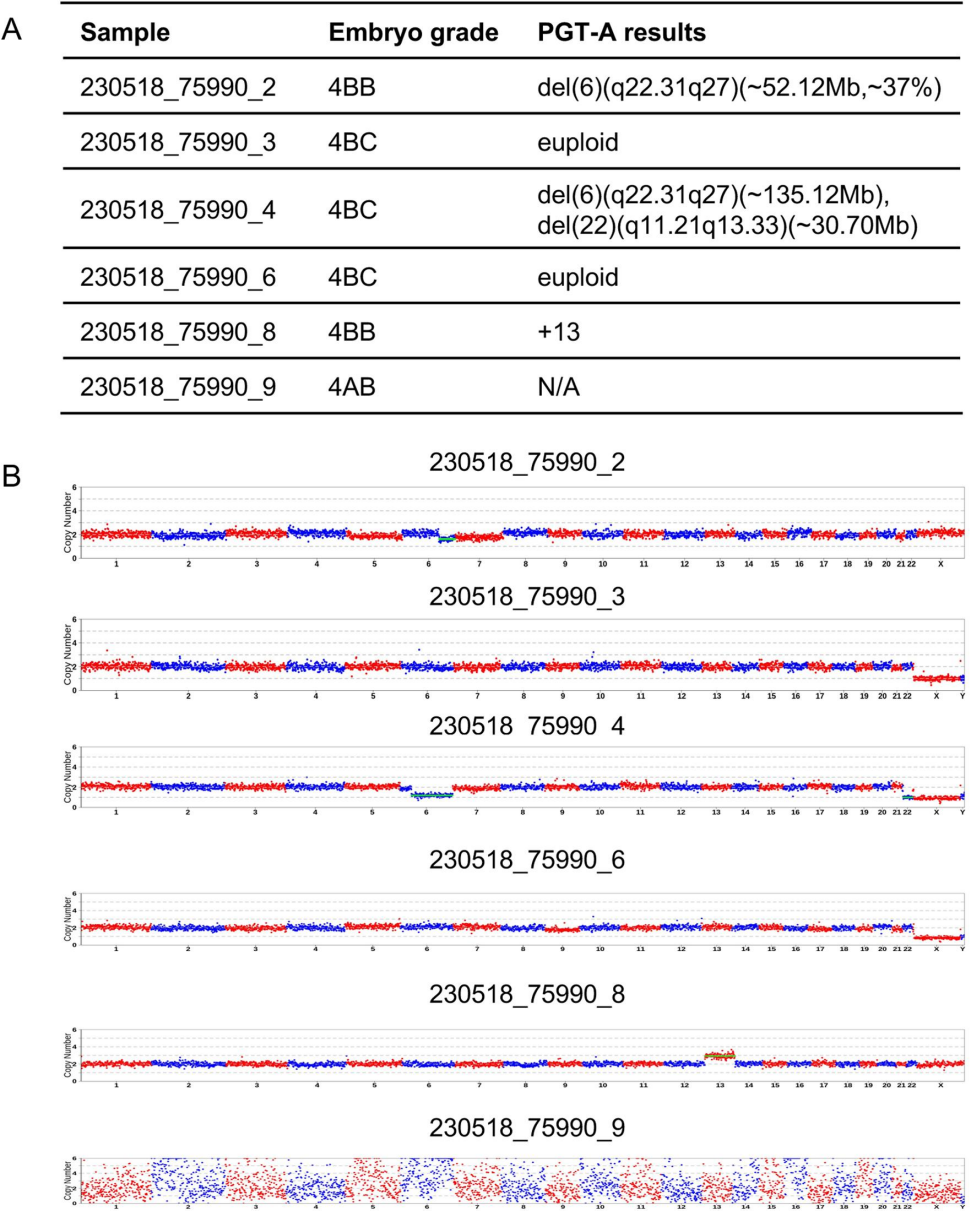
Genetic testing results of embryo and conduction of miscarriage in case 2. **(A)** PGT-A testing report. **(B)** Embryonic chromosomal copy number variations (CNVs) analysis profile.

**Figure 4:**
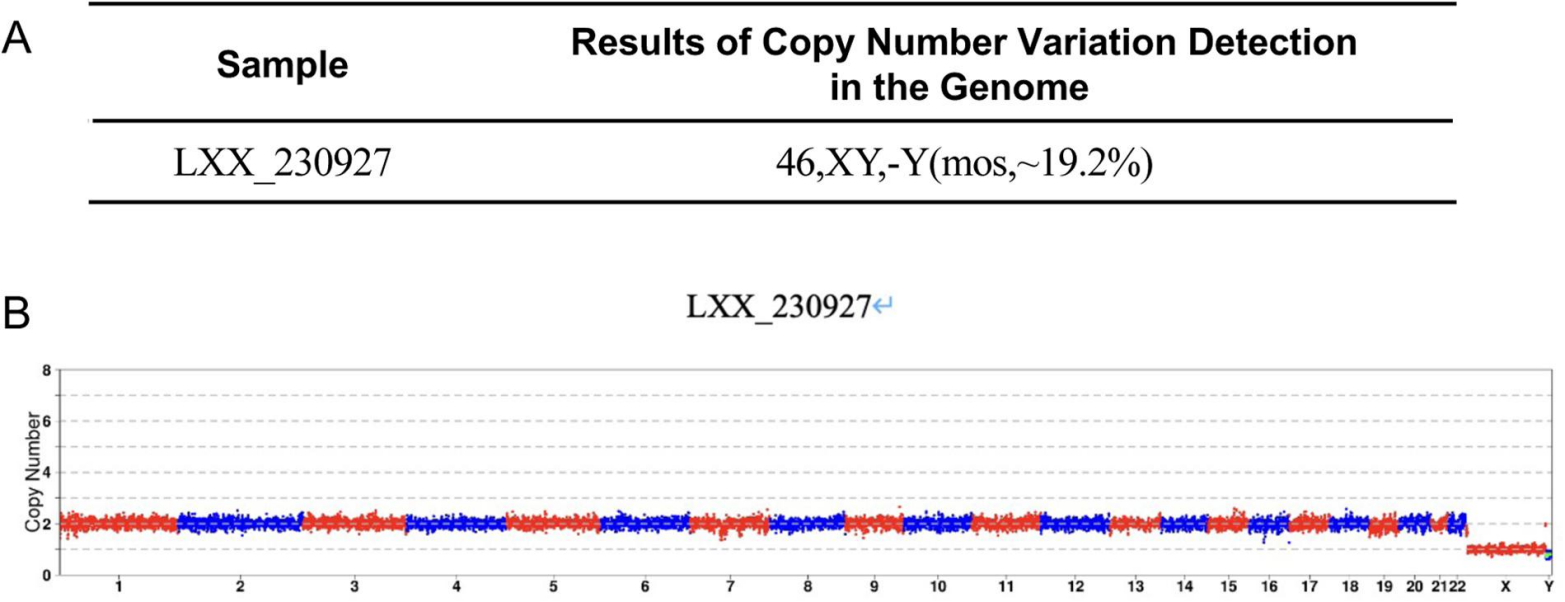
Genetic analysis of abortion tissue from Case 2. **(A)** Chromosomal copy number variations (CNVs) analysis report of abortion tissue. **(B)** CNVs analysis profile of abortion tissue.

Case 2: A 38-year-old female presented with a 2-year history of infertility. Hysterosalpingography indicated bilateral tubal patency with impaired filling. The semen analysis report showed no abnormalities. The patient's obstetric history included one missed abortion, for which dilation and curettage had been done. CNV analysis of the prior miscarriage tissue had revealed trisomy 16. The patient underwent PGT-A at our center with the primary indications of infertility and advanced maternal age. The use of an antagonist ovarian stimulation protocol resulted in the retrieval of 10 oocytes. Following ICSI, six blastocysts were successfully cultured and subsequently underwent trophectoderm biopsy. Biopsies of the trophectoderm followed by sequencing were undertaken using kits from XK and BGI, as previously mentioned. PGT-A results identified two euploid blastocysts, two aneuploid blastocysts, one suspected mosaic blastocyst, and one blastocyst with a non-informative result (Figure 3). Likewise, the woman was required to undergo a serum hCG test to exclude a pre-existing pregnancy. On August 3, 2023, one euploid blastocyst was thawed and transferred. Serum hCG level measured 12 days post-transfer was 1,871 mIU/mL. A transvaginal ultrasound at 44 days of pregnancy demonstrated a viable intrauterine singleton pregnancy. However, a follow-up ultrasound at 66 days revealed an intrauterine gestational sac containing an embryo without cardiac activity. After uterine evacuation, CNV analysis of the products of conception identified a 46,XY karyotype with mosaic loss of the Y chromosome (mos, ∼19.2%) (Figure 4).

## Discussion

Although PGT-A screens embryos for abnormal chromosome number before implantation, euploid embryos that are transferred can still lead to clinical pregnancy loss. Subsequent genetic analysis of the miscarriage tissue may reveal aneuploidy or mosaicism. This demonstrates the technical and biological limitations inherent to the PGT-A process. Multiple biological factors and technical issues might have resulted in this discrepancy.

PGT-A relies on the genetic analysis of 5–10 cells biopsied from the trophectoderm (TE), typically using high-throughput sequencing to assess CNVs. This process involves DNA fragmentation, amplification, and library construction using minimal cells. The resolution and scope of detection is intrinsically limited by the low starting material. Furthermore, reporting standards vary from different reproductive centers. The majority of those report deletions or duplications over 4 Mb and mosaicism only when the abnormal cell proportion exceeds a 20%–30% threshold. According to a fundamental biological constraint (3), the biopsy result of a little cells from TE could not be fully representative of the entire embryo or even the whole trophectoderm. Discrepancies in ploidy between the TE and the inner cell mass (ICM) can result in both false-positive (abnormal TE and normal ICM) and false-negative (normal TE and abnormal ICM) diagnoses. A previous study reported a high concordance rate (98.54%) when the euploid embryos are re-biopsied (4). Residual misdiagnosis risk is low but nonetheless clinically significant as demonstrated by the two cases, which occurred with 1.5% probability (4). The findings highlight the need for thorough and impartial patient counseling prior to PGT. Inadequate communication about these limitations can contribute to medical disputes regarding a subsequent miscarriage where an abnormal CNV result is detected in the pregnancy tissue. Moreover, the two cases show the indispensable values of prenatal diagnosis to the pregnancy after PGT-A treatments. As a results of the biological and technical limitation of PGT-A, embryos deemed euploid could still have a risk of mosaicism. One essential safeguard is prenatal diagnosis which identifies and manages the risk of birth defects possibly missed during preimplantation diagnostics.

Indeed, a spontaneous pregnancy could theoretically occur during the course of PGT-M treatment. In clinical practice, every patient is required to undergo a serum hCG test before preparing the endometrium for FET to exclude a pre-existing pregnancy. What's more, clinicians would advise couples to use contraception to avoid adverse pregnancy outcomes, such as miscarriage and giving birth to another child with a genetic disease. Therefore, in the current cases, the possibility that the mosaic miscarriage tissue did not originate from the transferred embryo is highly unlikely. In future studies, DNA fingerprinting analysis could be considered to confirm the one-to-one correspondence between miscarriage tissue and the transferred embryo.

In the two cases presented herein, the embryos that were initially considered euploid according to a trophectoderm biopsy were found to be mosaic on analysis of miscarriage issue. It's reported that the prevalence of NGS-detected mosaicism at the blastocyst stage range from 6.6% to 29.1% (5–7). Several factors are implicated in the occurrence of mosaicism, including delayed blastocyst development, poor blastocyst quality, semen parameters, and advanced paternal age. Besides, it should also be noted that paternal factors, such as elevated sperm DNA fragmentation or sperm chromosomal aneuploidy, may exert a potential influence on the formation of embryonic mosaicism. Such defects may compromise early embryonic development by disrupting post-zygotic mitotic fidelity, thereby increasing the likelihood of mosaicism formation and subsequent pregnancy loss. In Case 2, analysis of the miscarriage tissue revealed the presence of a sex chromosome abnormality, suggesting a potential contribution from the paternal genome. Furthermore, it's possible that the different biopsy protocol used may have an impact on the reported mosaicism rate (8–10). It is a known fact that the distinct cell lineages in mosaic embryo are often unevenly distributed. A recent study conducted by Kim et al. on re-biopsy of mosaic blastocysts from four different sites reported concordance rates of whole-chromosome mosaicism and segmental mosaicism as low as 39.08% and 41.94%, respectively (4). The inconsistent results between the initial PGT-A report and the CNV analysis of different parts of the abortion tissue in the first case further supports the low concordance of mosaic embryos. Since the first report by Greco et al. of a successful live birth following the transfer of a mosaic embryo (11), many such cases have been published. However, it has been reported that transferring mosaic embryos is associated with lower implantation rates, fewer live births and increasing miscarriage rates, compared to the transfer of euploid embryos (12, 13). In addition, it's reported that transfers of embryos diagnosed as mosaic have resulted in live births, a subset of which have confirmed the presence of the mosaicism or aneuploidy in the newborn (14–17). In light of this evidence and the latest expert consensus on mosaic embryos (18, 19), the clinical counselling of mosaic embryo transfer should be optimistic but cautious.

## Data Availability

The original contributions presented in the study are included in the article/Supplementary Material, further inquiries can be directed to the corresponding author.
